# The Relationship between Protein Intake and Lean Mass in Non-Hispanic Black and East Asian Females

**DOI:** 10.21203/rs.3.rs-9621184/v1

**Published:** 2026-06-10

**Authors:** Evan Levy, Keanu Lettley, Nevaeh Nez, Guillermo Pacheco, Malia N. M. Blue

**Affiliations:** University of North Carolina at Chapel Hill; Triangle Arthritis and Rheumatology Associates; University of Colorado Anschutz Medical Campus; University of North Carolina at Chapel Hill; University of North Carolina at Chapel Hill

**Keywords:** Macronutrient Intake, Fat Free Mass, Minority Health

## Abstract

**Background:**

Low lean mass (LM) is associated with increased cardiometabolic disease risk and increased injury risk. Protein (PRO) intake is an important strategy to build and maintain LM. There is limited data quantifying PRO intake and evaluating the association between PRO intake and LM in non-athletic, young adult, female populations, and even less in females of color. The purpose of this study was to characterize PRO intake and evaluate the association between PRO intake and LM in females of color.

**Methods:**

85 Non-Hispanic Black (n=42) and East Asian (n=43) females (Age: 22.5 ± 3.8 yrs, BMI: 23.8 ± 3.5 kg/m^2^) completed this study. Participants completed a 3-day dietary log. A dietary analysis software assessed total caloric intake and total PRO intake and PRO intake as a percentage of total calories (%PRO). LM was assessed using dual-energy x-ray absorptiometry. Multiple linear regression analyses were performed to assess the association between PRO intake and LM, after controlling for potential cofounding variables (e.g., race, age, MVPA).

**Results:**

For the full sample, PRO intake was 86.1 ± 30.0 g and %PRO was 17.4 ± 4.8%. After accounting for covariates, there were significant correlations between total PRO intake (g) and LM (p < 0.001, Δr^2^ = 0.304) and %PRO and LM (p = < 0.001, Δr^2^ = 0.232). PRO intake was a significant predictor of LM in NH Black (p = 0.008) and East Asian females (p = 0.005); %PRO was a significant predictor of LM for NH Black females (p = 0.035) only.

**Conclusions:**

In this ethnically diverse sample of young-adult females, PRO intake and %PRO were within recommended guidelines to support LM. We found a moderate association between total PRO intake and LM in both ethnicities; however, %PRO may be a more important metric of adequate protein intake to support LM in NH Black females.

## Background

Diet-related diseases such as type 2 diabetes (T2D), heart disease, and stroke, have a higher prevalence in racial minority populations in the U.S. (1). Non-Hispanic (NH) Black individuals experience the highest prevalence of diet-related health disparities when compared to other racial and ethnic groups. Specifically, according to 2011–2018 NHANES data, NH Black populations had a higher prevalence of T2D (26.8%), obesity (50.2%), hypertension (59.3%), and lower ideal diet score prevalence (8.2%) based upon the healthy eating index (HEI) when compared to NH White, Other Hispanic, and NH Asian populations (2). Although Asian individuals residing in the U.S. demonstrated a higher ideal diet score prevalence (20.9%) ((2) and lower rates of obesity (12.1%), in large national studies (e.g., MESA, MASALA), East Asian and South Asian groups demonstrated higher rates of metabolic abnormalities at normal weight BMIs when compared to NH White populations (3)(4) Further, Asian populations demonstrate a higher propensity for visceral and hepatic fat storage, and lower lean mass (LM) compared with other ethnic populations (5, 6).

Adequate protein (PRO) intake may have a protective effect for cardiometabolic diseases (7). Protein intake is critical for muscle protein synthesis and LM preservation and growth (8). LM releases myokines during contraction which have important endocrine and metabolic effects (9). Data on PRO intake and LM is limited in college-aged NH Black and East Asian females, especially in non-athletic populations. However, previous literature does demonstrate there may be differences in LM between East Asian and NH Black females, possibly indicating different PRO intake requirements and characteristics (10).

Contrary to prior studies in predominantly NH White individuals, a study by Popp et al. in 2019 (11) found that PRO intake as a percentage of total caloric intake (%PRO) in Asian American individuals was positively correlated with adiposity. Additionally, a cross-sectional study of NHANES 2011–2016 data found a negative correlation between PRO intake and LM in a multiethnic cohort of adult females, but a positive correlation between PRO quality and physical activity with LM (12). This paradoxical relationship between PRO intake and body composition characteristics suggests further research is needed to understand PRO intake and body composition in these populations, and the subsequent impact on cardiometabolic disease. Further, a systematic review examining nutritional health intervention studies observed that only 2% reported and discussed health outcomes in multiple racial or ethnic groups and 1.5% studied a single racial or ethnic group (13). This data highlights the need for further diet and health research in both NH Black and Asian female populations. Therefore, the purpose of this study was to characterize PRO intake and evaluate its association with LM in NH Black and East Asian females. We hypothesized that both total PRO intake and %PRO will be positively associated with LM in both NH Black and East Asian females.

## Methods

### Study Design

For this cross-sectional study, participants were asked to report to the laboratory for one testing session. Participants were at least 8 hours fasted including no caffeine, tobacco, or alcohol, and they had refrained from strenuous exercise for at least 24 hours. All participants completed a written informed consent approved by the University’s Institutional Review Board prior to their participation in this study. Following enrollment, a member of the research team measured height and weight, followed by a urine HCG pregnancy test. Body composition was measured by dual energy x-ray absorptiometry (DXA). Dietary intake was evaluated by a 3-day dietary log. Additionally, participants completed a series of lifestyle questionnaires to assess physical activity, stress, and social determinants of health.

### Participants

The proposed study enrolled 90 healthy females who self-identified as non-Hispanic (NH) Black (n=45) and East Asian (n=45). The full inclusion criteria for eligibility were: 1) free of any pre-existing cardiometabolic or musculoskeletal disorders, 2) self-identify as female, 3) self-identify as NH Black or East Asian and have parents of the same race, 5) body mass index between 18.5 to 34.9 kg/m^2^. We excluded participants if they: 1) were taking medicine that may alter body composition, 2) were currently pregnant or planning to become pregnant, 3) had lost or gained more than 3 kilograms in the previous 2 months, 4) recently started, stopped or changed exercise habits (e.g., resistance training to endurance training), 5) clinically or self-diagnosed with an eating disorder, 5) were completing hormone therapy (to change biological sex).

### Measures

#### Dietary Intake

Dietary intake was assessed by a 3-day dietary log which was completed within a week of the study visit. Participants were asked to record all food and beverage consumption for two weekdays and one weekend day. A research team member reviewed the 3-day diet log upon arrival for clarity and to confirm details of diet. Specifically, these details included the date, name or brand of food and beverage, the amount consumed and confirming all meals and snacks had been included. Intake was entered into MacroFactor Software (Version 2.1.5 [246], Stronger by Science Technologies, LLC, NC, USA), a newly developed dietary app which has a food database that includes 1,360,000 verified food items. Total daily calorie intake and total protein intake (g) were recorded. PRO intake as a percentage of total calories (%PRO) was calculated by [PROgrams×4calories]/Totalcalories.

### Body Composition

A total body dual energy x-ray absorptiometry (DXA) (GE Lunar iDXA, Madison, WI, USA) scan was completed and analyzed by a research team member to determine %fat, and total body LM. Prior to testing, participant information, including date of birth, height, weight, ethnicity and sex was entered into the DXA software. Additionally, participants were instructed to remove shoes, all metal (e.g., jewelry, clothing with zippers) and heavy plastic or thick material (e.g., sweatpants/sweatshirt). Participants were then placed in the center of the DXA scanning table. To minimize the influence of position on DXA measures, a research team member ensured the participant is aligned by placing Styrofoam pads between the participants hands and thighs and on the participants feet to keep them in the dorsiflexed position. Participants were instructed to remain still for the duration of the scan. Regions-of-interest were manually adjusted by a trained member of the research team.

### Lifestyle Factors

#### Physical Activity

Physical activity (PA) was measured using the validated International Physical Activity Questionnaire (IPAQ, Short Form), a survey designed to assess PA among various populations (14). Participants answered five questions to self-report their time spent in physical activity in the previous 7 days. Specifically, participants were asked to provide the days per week and minutes per day spent in activities such as walking, participating in moderate and vigorous physical activity (MVPA) as well as sedentary activity.

### Statistical Analysis

Mean, standard deviation and frequency data were calculated for continuous and categorical variables, respectively. Separate stepwise linear regression analyses were performed to assess the association between LM and PRO intake, and LM and %PRO controlling for potential cofounding variables (e.g., race, age, MVPA). If baseline characteristics were not found to significantly contribute to the model, they were excluded and linear regression analysis results were reported. Regression analyses were completed in the full sample, and in each race separately. All statistical analyses were conducted using IBM SPSS Statistics (Version 28) with an a priori alpha level of p ≤ 0.05.

### Results

Participant descriptive characteristics are presented in Table 1. Of the 90 individuals enrolled, 5 individuals were removed from analyses (n = 3, missing dietary data, n = 2, missing physical activity data), therefore data from 85 individuals were analyzed (n = 42 NH Black, n = 43 East Asian). There were no statistically significant differences for MVPA or protein intake between races; NH Black participants had greater BMI (p < 0.001) and LM compared to East Asian participants (p < 0.001).

Results of the stepwise linear regression analyses evaluating the association of total PRO and %PRO with LM, while controlling for age, MVPA, and race are shown in Table 2. Race was significant and included in both models, while MVPA and age were not significant and were excluded from both models. Both PRO intake (b = 0.072, t(82) = 4.0, p < 0.001) and %PRO (b = 0.303, t(82) = 2.597, p = 0.011) were significant predictors of LM. ΔR^2^ represents the change in variance in LM explained after adding PRO or %PRO into models 1 or 2, respectively.

Results of the stepwise linear regression analyses performed in each race are presented in Table 3. Age and MVPA were not significant and therefore were excluded from the models. Each 10g increase in PRO intake predicted a LM increase of 0.39 kg in NH Black individuals (b = 0.086, t(40) = 2.768, p = 0.008) and a 0.28kg increase in East Asian Individuals (b = 0.061, t(41) = 3.004, p = 0.005).

Each 1% increase in %PRO predicted a LM increase of 0.18 kg for NH Black individuals (b = 0.390, t(40) = 2.180, p = 0.035), but %PRO was not a significant predictor for East Asian individuals (b = 0.203, t(41) = 1.361, p = 0.181).

## Discussion

The purpose of this study was to characterize PRO intake and evaluate its association with LM in NH Black and East Asian females as previous literature in multi-ethnic samples have reported inconsistent associations between protein intake and body composition. Further, research specifically examining this relationship in college-age NH Black and East Asian females is lacking. In this sample, we found a significant positive association between total PRO intake and LM. PRO intake explained 16.1% and 18% of the variance in LM for NH Black and East Asian individuals, respectively. %PRO explained 10.6% of variance in LM in NH Black females but was not a significant predictor of LM in East Asian females. We found a significantly lower LM (38.7 ± 4.5kg) in East Asian females compared to NH Black females (43.4 ± 6.0kg), with similar levels of physical activity. While previous literature exists demonstrating that PRO intake above the daily recommended dietary allowance (RDA) of 0.8g/kg supports increases in LM in healthy adults with or without resistance training, there is minimal data available in people of color or in college-age females, and the available literature is not conclusive.

In opposition to our findings, a study by Xu et al. in 2020(12) found a negative association (b = −0.84, p < 0.001) of PRO and LM in middle-aged NH Black, NH White, and Hispanic females with PRO intakes (mean = 1.1g/kg/day), higher than the RDA. In a sample of middle-aged Chinese American males and females (52.9 ± 14 years), %PRO intake was negatively associated with relative percent LM and not associated with total LM. This negative relationship was observed even though the Chinese Americans had a significantly higher relative protein intake (1.34g/kg body weight per day) at roughly 19.2% of energy intake compared to the general population at 15% of energy intake(11) However, similar to our study findings, the Health, Aging, and Body Composition (Health ABC) longitudinal study reported a positive relationship between PRO intake and LM in NH Black females (15). While this study was in an elderly population (70–79 yrs), the results showing that higher baseline PRO intake was associated with decreased loss in appendicular LM(15) demonstrates the importance of PRO intake for LM preservation with aging.

Literature describing the relationship between PRO and LM in college-age female populations often does not report race or ethnicity and focuses on athletic populations mass (16, 17). Two studies in college-age female athletes found PRO supplementation was associated with maintenance of LM and reduced fat mass (16, 17), but neither study reported race or ethnicity of participants. A.F Brown et al. (16) found in a 12-week study that female collegiate dancers consuming whey protein at 2.2 g/kg/day preserved or slightly increased LM, while a placebo group consuming 0.9 g/kg/day lost around 1.1kg of lean mass. Wilborn et al. (17) looked at LM response to an added 24g of protein supplementation both before and after practices 4 times per week in 16 NCAA female basketball athletes and found an increase in LM of 1.4 ± 1.0kg and 1.5 ± 1.0kg for whey protein and casein protein supplementation groups, respectively. These findings support the potential benefit of increased protein intakes within college-age females.

A meta-analysis by Tagawa et al. in 2021(18) including 5402 male and female participants from 105 articles found a positive association between PRO intake and LM for PRO intakes up to 3.5g/kg/day, with a pronounced effect of PRO intake up to 1.3g/kg/day. This data(18) was drawn primarily from NH White participants (91/105 studies included primarily NH White participants) ranging in mean age from 18 to 81 years with an overall mean age of 47.2 years. Our results are consistent with the findings in college athlete literature (16, 17) and the general population (18), supporting PRO intakes above the RDA for maintenance of LM in both NH Black and East Asian college-age females. Both our NH Black and East Asian groups had relative protein intakes of 1.3 ± 0.4g/kg/day, which is above the RDA of 0.8g/kg/day and aligns with the findings of Tagawa et al. (18), suggesting a protein intake of ≥ 1.3g/kg/day may be necessary to see the positive association between PRO intake and LM.

The %PRO intake of our East Asian participants (17.2 ± 4.6%) was consistent with NHANES 2011–2016 data (17.3%), but %PRO intake of our NH Black participants (17.6 ± 5.1%) was higher than %PRO intake reported by NHANES (15.3%) (19). This 2011–2016 report found that Asian Americans had the highest relative protein intake compared to NH White, NH Black, and Hispanic participants. NH Black individuals had the lowest relative intake (19). The Acceptable Macronutrient Distribution Range (AMDR) is 10–35% of caloric intake, with evidence supporting an optimal intake of ≥ 18% for LM maintenance(20). Our findings showed %PRO intake as one of the predictors of LM in our sample of NH Black females, but not East Asian females. %PRO may be an important metric of adequate PRO intake in NH Black females due to higher LM and therefore higher PRO recommendations to maintain mass. One additional consideration for this study sample is their overall energy balance. While outside the scope of this paper, based on reported PA levels and sex-predicted total daily energy expenditure (21). The NH Black group had a higher caloric intake and a lower predicted caloric deficit. The East Asian group had a lower caloric intake and greater predicted deficit, meaning higher %PRO in a low-calorie diet may still be inadequate protein intake to influence LM.

Although this study adds to the existing literature by including an underrepresented group (e.g., college-age females of color), there are limitations to address. The present study relied on self-reported data from individuals from a single region with high educational attainment. Sampling from a highly educated area may bias our participants towards higher protein intakes and MVPA when compared to areas of lower educational attainment and socioeconomic status. Additionally, our findings in East Asian females may not be applicable to females within other Asian subgroups; there is variability in cultural dietary patterns and body composition norms between the large number of Asian ethnic groups. Further research including multiple Asian ethnic populations and multiple geographic locations could improve the generalizability of PRO intake associations. Future studies may also benefit from examining the associations between specific protein sources and protein quality with LM in college-aged females of color.

## Conclusions

In this study of college-aged NH Black and East Asian females, we found a significant association between total PRO intake and LM, supporting a protein intake of ≥ 1.3g/kg/day for the maintenance of LM in these populations. %PRO of total energy intake showed a significant association with LM in NH Black females, but not East Asian females; this suggests %PRO of total energy intake may be an important metric of adequate protein intake for NH Black females specifically due to higher average caloric intake and higher LM. Further research exploring PRO intake and LM while considering total energy balance in active, college-aged females of color is needed to better characterize PRO intake and understand optimal PRO intake in these understudied populations.

## Figures and Tables

**Table 1:** Mean ± standard deviation (SD) of participant characteristics and protein intake.

Total	n	Age (yrs)	BMI (kg/m^2^)	MVPA (min/wk)	Lean Mass (kg)	Protein (g)	Total Calories (kcal/day)	%Protein (%)
	85	22.5 ± 3.8	23.8 ± 3.5	137.0 ± 130.7	41.0 ± 5.8	86.1 ± 30.0	2017.8 ± 542.2	17.4 ± 4.8
NH Black	42	22.8 ± 4.1	25.5 ± 3.6*	131.6 ± 134.9	43.4 ± 6.0*	90.7 ± 28.2	2113.0 ± 567.3	17.6 ± 5.1
East Asian	43	22.2 ± 3.6	22.1 ± 2.6*	142.3 ± 127.7	38.7 ± 4.5*	81.5 ± 31.4	1892.2 ± 479.3	17.2 ± 4.6

BMI: body mass index, MVPA: moderate-to-vigorous physical activity.

* Denotes significant difference between NH Black and East Asian groups at p < 0.05. %Protein = Protein expressed as percentage of total calories

**Table 2:** Regression analyses for total protein and protein percentage of total calories and lean mass.

Model	R^2^	ΔR^2^	F	p
Total PRO	.552	.304	17.925	< 0.001*
%PRO	.481	.232	12.359	< 0.001*

Both models controlled for race.

* Denotes significance at p < 0.05. Total protein intake = PRO; protein percentage of total calories = %PRO.

**Table 3:** Simple regression analyses for total protein intake, protein percentage of total calories, and lean mass by race.

Race	R^2^	F	p
Total PRO			
NH Black	.161	7.663	.008*
East Asian	.180	9.027	.005*
%PRO			
NH Black	.106	4.752	.035*
East Asian	.043	1.854	.181

* Denotes significance at p < 0.05. Total protein intake = PRO; protein percentage of total calories = %PRO; Non-Hispanic = NH

## Data Availability

Data used for this study will be made available on reasonable request from the corresponding author.

## Abbreviations

AMDR: Acceptable macronutrient distribution range
BMI: Body mass index
DXA: Dual energy x-ray absorptiometry
IPAQ: International physical activity questionnaire
LM: Lean mass
MASALA: Mediators of atherosclerosis in South Asians living in America
MESA: Multi-ethnic study of atherosclerosis
MVPA: Moderate to vigorous physical activity
NHANES: National health and nutrition examination survey
NH: Non-Hispanic
PA: Physical activity
PRO: Protein
T2D: Type 2 diabetes
%PRO: Protein percentage of total caloric intake
